# Supplementary figures and images for: Genomic profiling and expression analysis of the diacylglycerol kinase gene family in heterologous hexaploid wheat
Source: PeerJ. 2021 Dec 14;9:e12480. doi: 10.7717/peerj.12480 (PMC8679913; doi:10.7717/peerj.12480)

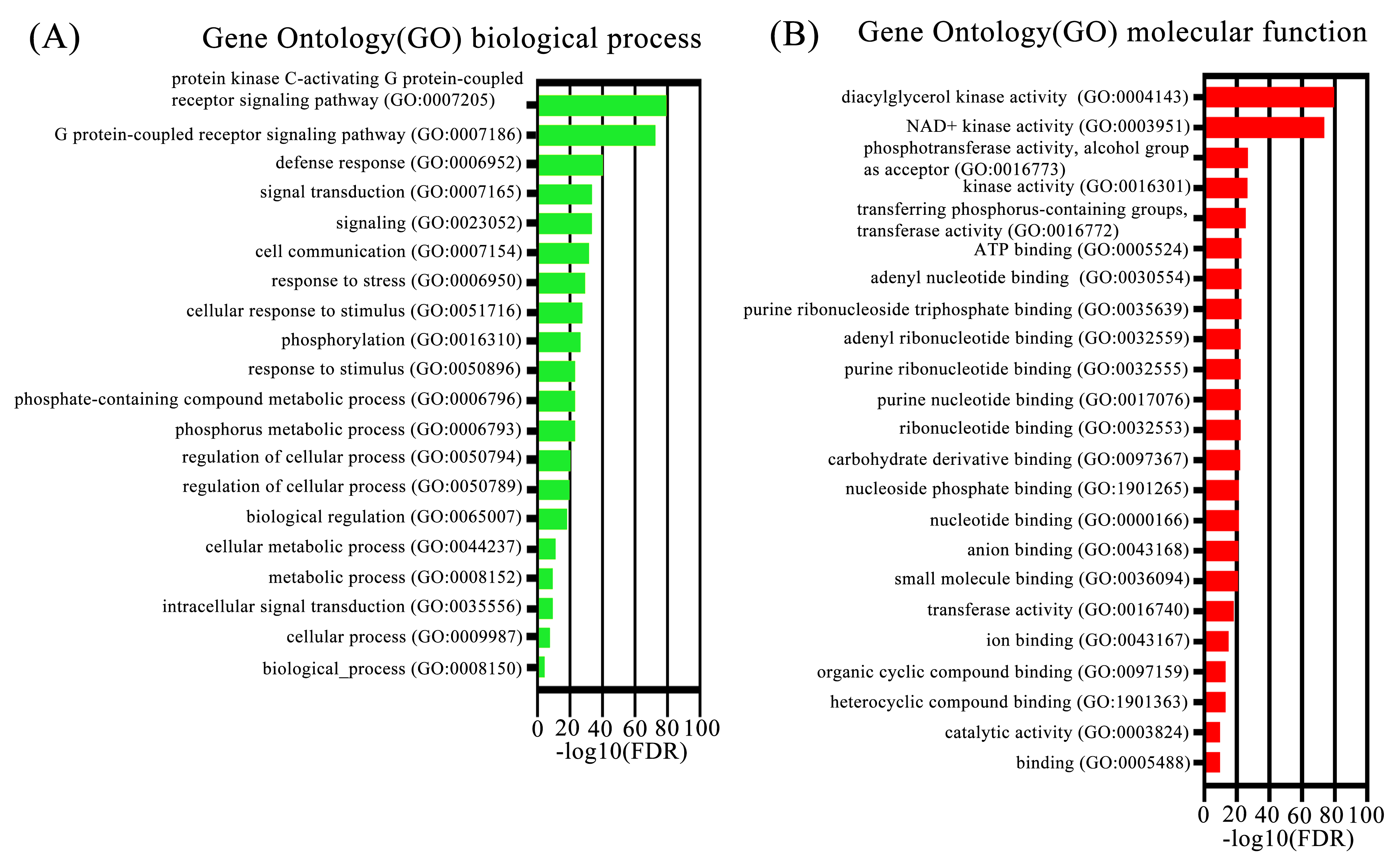

Supplement: Supplemental Information 1 [file peerj-09-12480-s001.png]

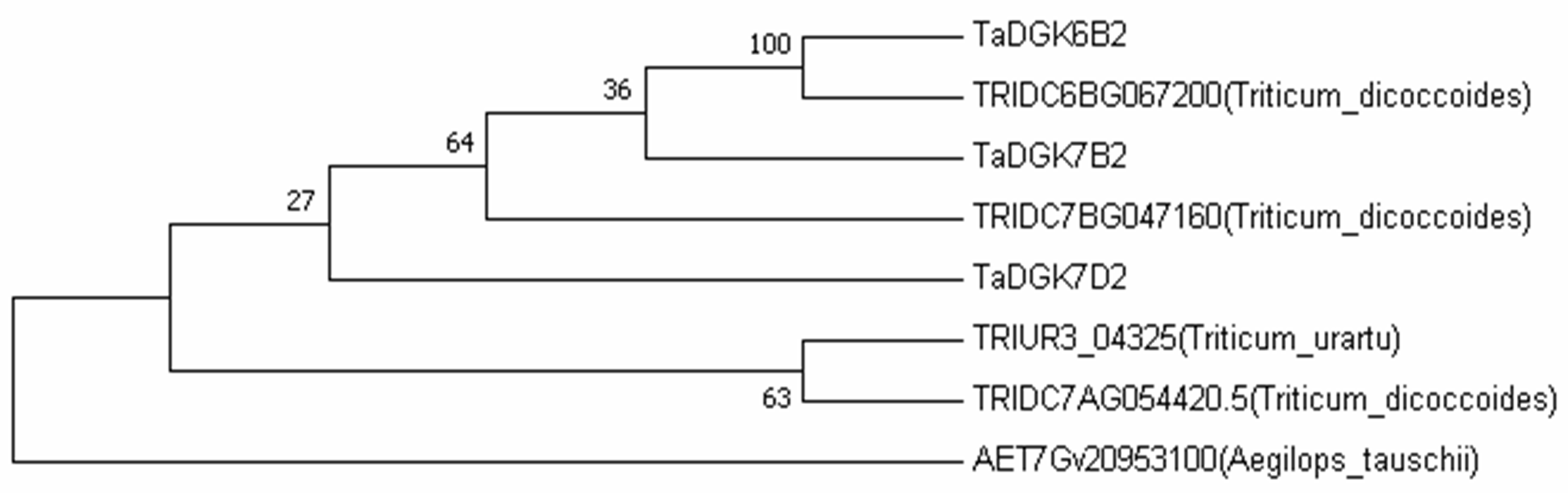

Supplement: Supplemental Information 2 — The numbers beside the branches indicate the bootstrapvalues that support the adjacent nodes. [file peerj-09-12480-s002.png]

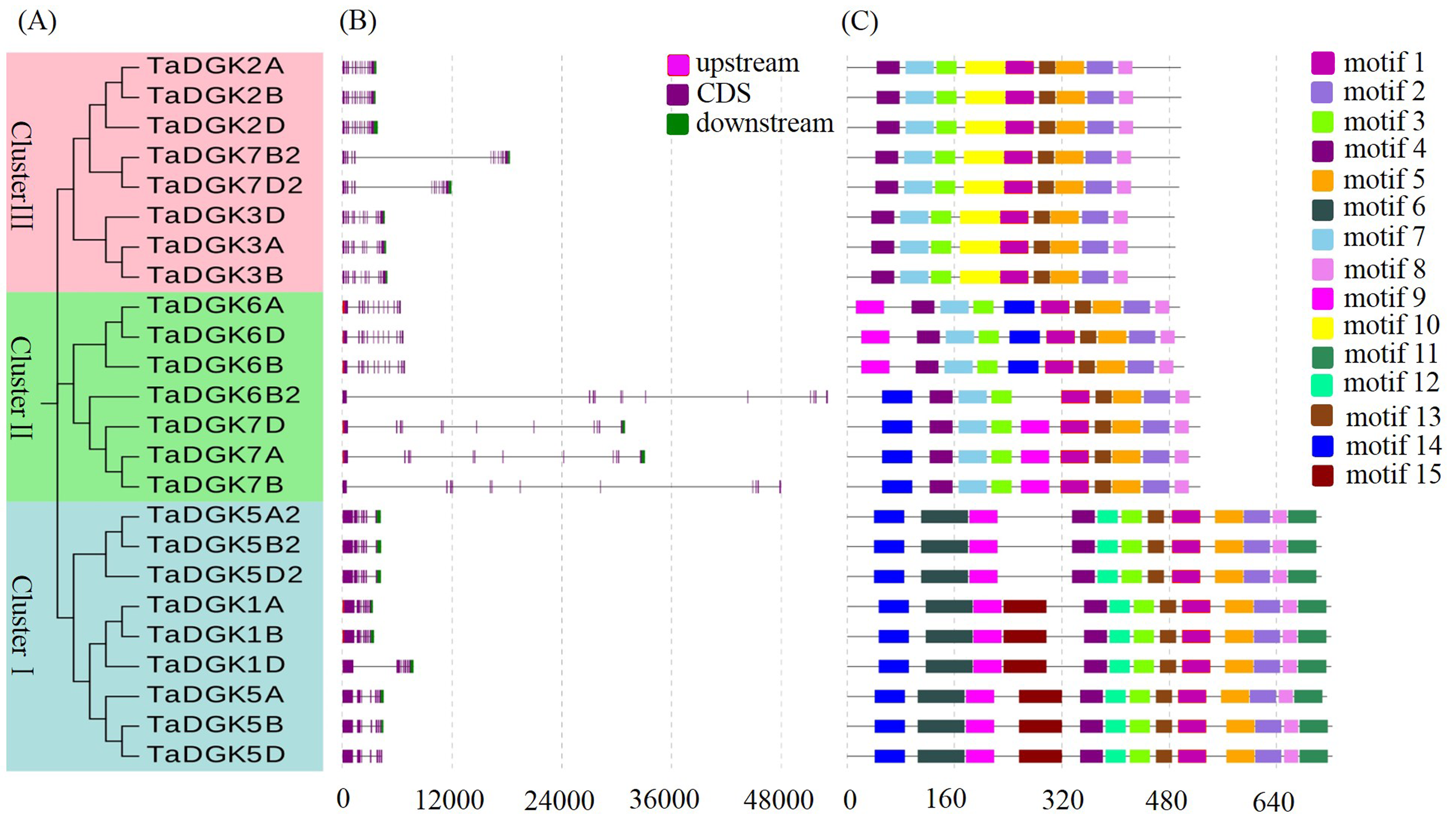

Supplement: Supplemental Information 3 — (A) Phylogenetic analysis, with different colors indicating genes in individual clusters. (B) Schematic diagram of exon/intron structures of DGK genes in rice and wheat. Red, purple, and green boxes represent upstream regions, CDSs, and downstream regions, respectively. (C) Schematic diagram of the DGK gene motifs. Different colored boxes are assigned to each motif. [file peerj-09-12480-s003.png]

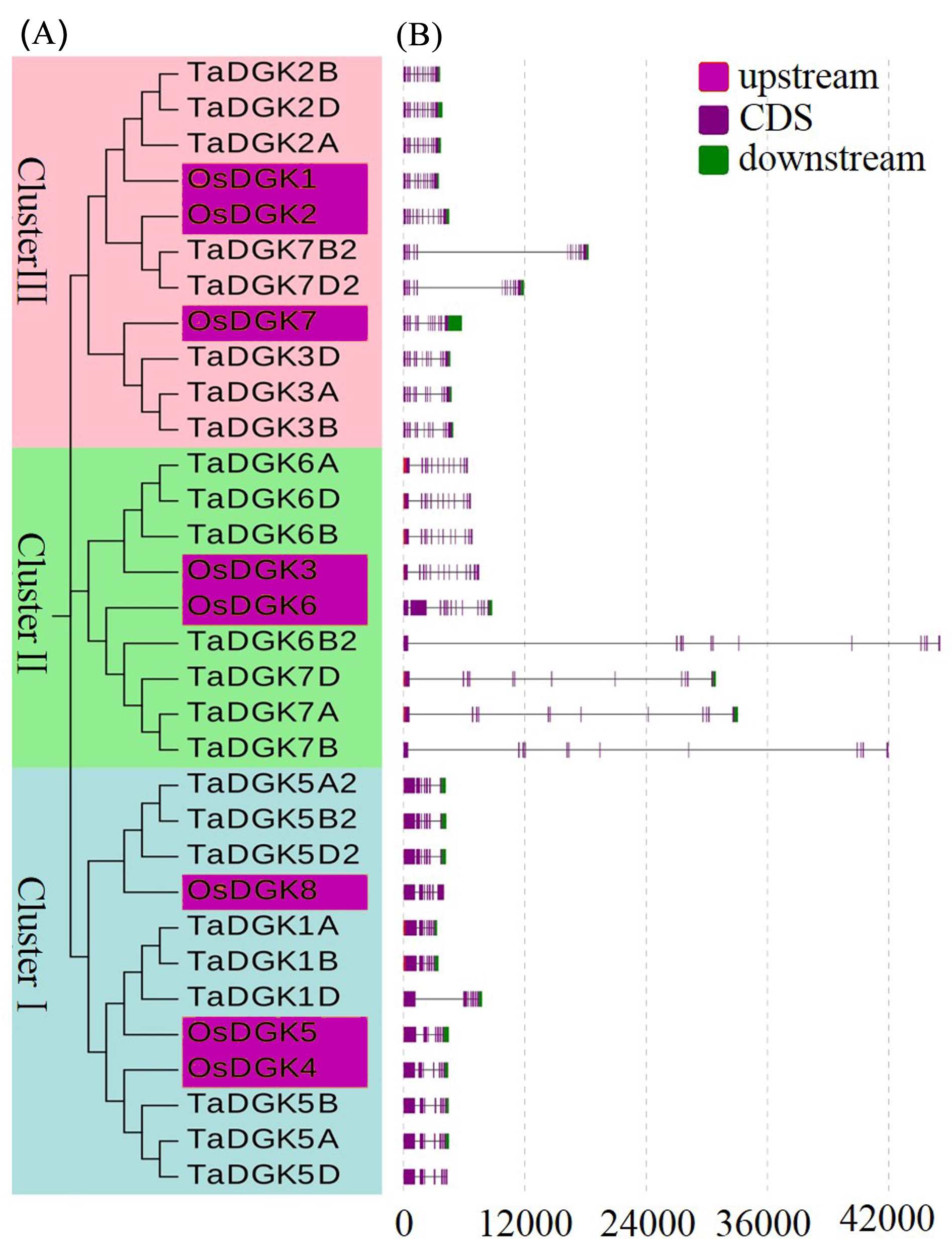

Supplement: Supplemental Information 4 — (A) Phylogenetic analysis, with different colors indicating genes in individual clusters. (B) Schematic diagram for exon/intron structures of DGK genes in rice and wheat. Red, purple, and green boxes represent upstream regions, CDSs, and downstream regions, respectively. [file peerj-09-12480-s004.png]

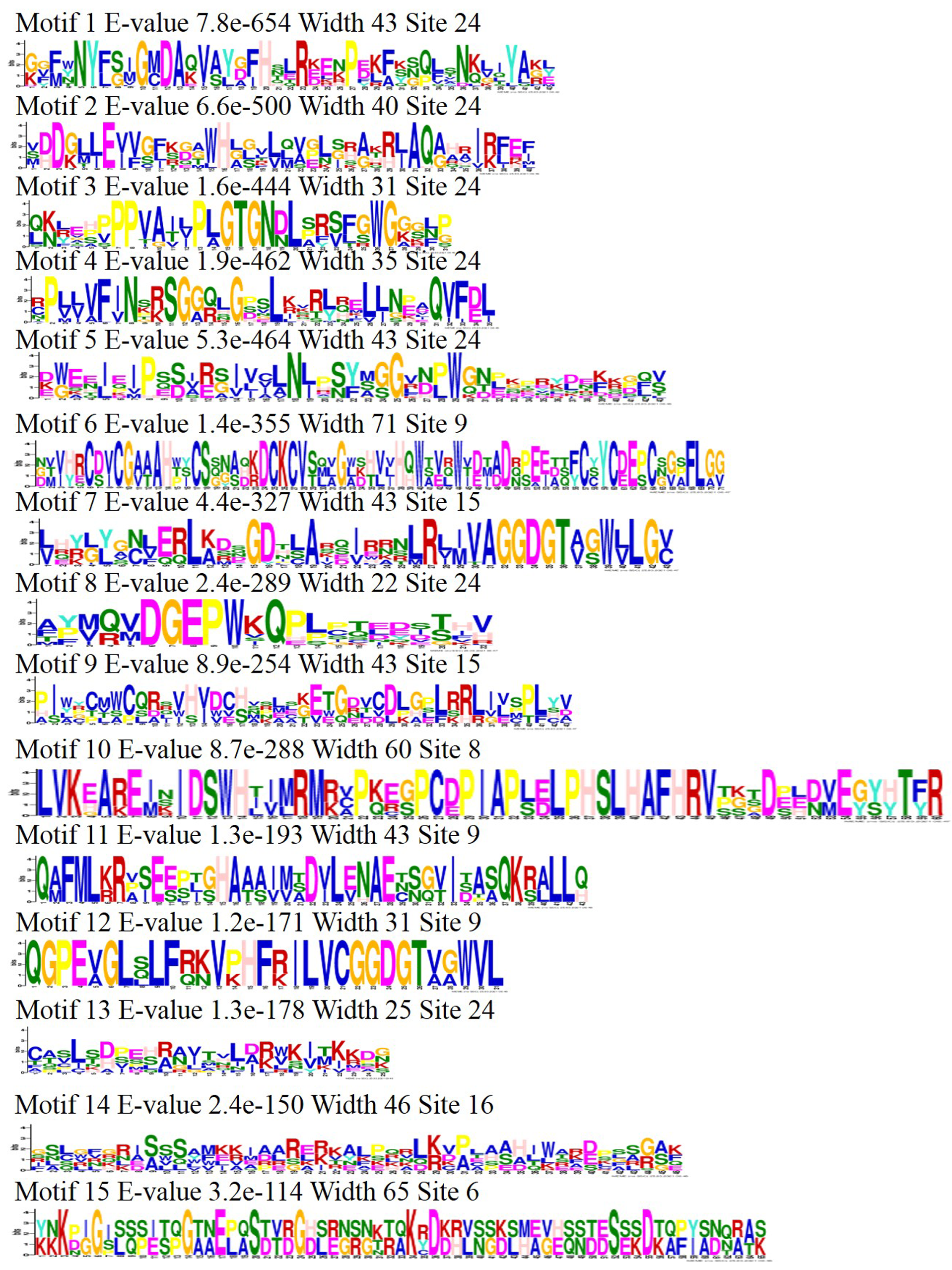

Supplement: Supplemental Information 5 [file peerj-09-12480-s005.png]

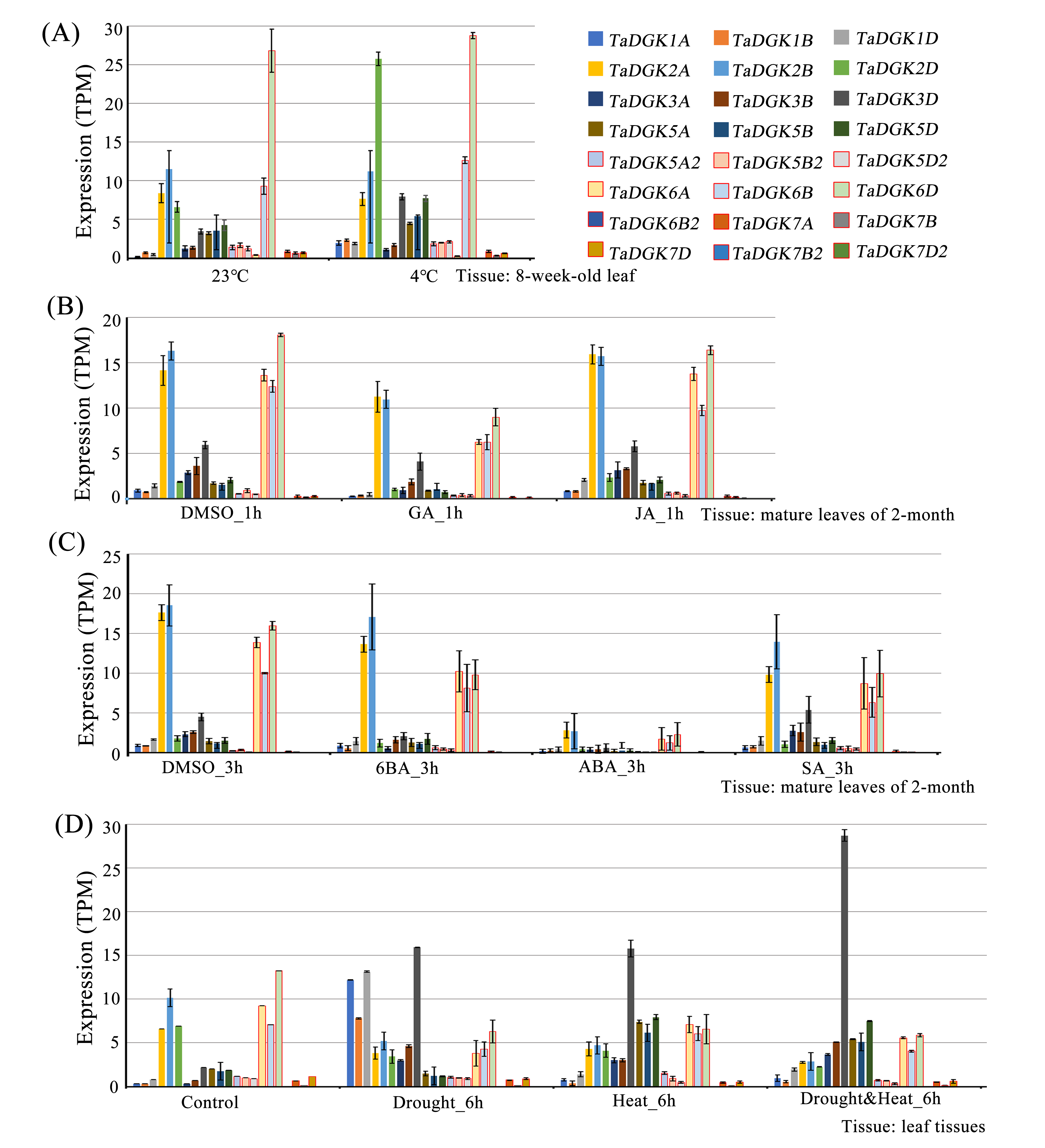

Supplement: Supplemental Information 6 [file peerj-09-12480-s006.png]

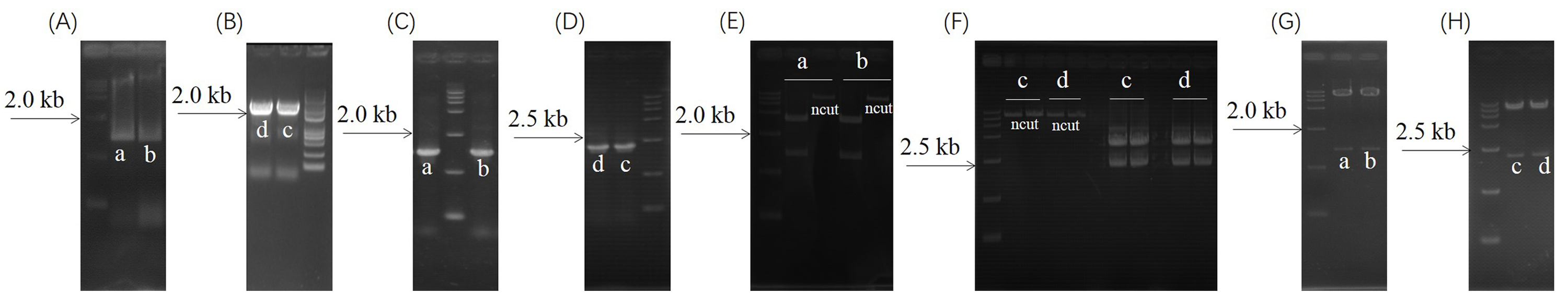

Supplement: Supplemental Information 7 — Subcellular localization vector construction for (a) 35S:TaDGK2A-GFP, (b) 35S:TaDGK3A-GFP, (c) 35S:TaDGK5B-GFP, and (d) 35S:TaDGK5A2-GFP. (A) Amplification of TaDGK2A CDS and TaDGK3A CDSs. (B) Amplification of TaDGK5B CDS and TaDGK5A2 CDS. (C) Colony PCR verification of pEASY-TaDGK2A and pEASY-TaDGK3A constructs. (D) Colony PCR verification of pEASY-TaDGK5B and pEASY-TaDGK5A2 contructs. (E). Double enzyme SpeI/KpnI digest for pEASY- TaDGK2A; Double enzyme XbaI/KpnI digest for pEASY-TaDGK3A. (F). Double enzyme SalI/SpeI digest for pEASY-TaDGK5B; double enzyme Xba I/Kpn I digest for pEASY-TaDGK5A2. (G). Double enzyme SpeI/KpnI digest for 35S:TaDGK2A-GFP; double enzyme Xba I/Kpn I digest for 35S:TaDGK3A-GFP. (H). Double enzyme SalI/SpeI digest for 35S:TaDGK5B-GFP; double enzyme XbaI/KpnI digest for 35S:TaDGK5A2-GFP. [file peerj-09-12480-s007.png]
